# Charity Begins at Home: Understanding the Role of Corporate Social Responsibility and Human Resource Practices on Employees’ Attitudes During COVID-19 in the Hospitality Sector

**DOI:** 10.3389/fpsyg.2022.828524

**Published:** 2022-02-22

**Authors:** Albert John, Gulnaz Shahzadi, Kanwal Iqbal Khan, Shafaq Chaudhry, Muhammad Arslan Sarwar Bhatti

**Affiliations:** ^1^Lahore Business School, University of Lahore, Lahore, Pakistan; ^2^National College of Business Administration and Economics, Lahore, Pakistan; ^3^Institute of Business and Management, University of Engineering and Technology, Lahore, Pakistan

**Keywords:** CSR, HR practice, organizational identification, employees commitment, sensemaking theory

## Abstract

The COVID-19 outbreak wreaked havoc on the hospitality business, resulting in significant layoffs, salary cuts, and unpaid leaves globally. This study uses the sensemaking theory to investigate how COVID-19 induced unfavorable human resource (HR) practices affect the link between perceived corporate social responsibility (CSR) and employee identification and commitment. We tested this model using the data collected from 392 hospitality sector employees in Pakistan. The results reveal that “cut in salaries” and “work from home” positively moderate CSR’s impact on employees’ identification and commitment. On the other hand, employee layoff and leave without pay do not impact the positive relationship between CSR and employees’ attitudes. Furthermore, the study finds that CSR during this pandemic has a significant positive impact on employees’ attitudes. However, this relationship becomes insignificant for employees who reported unfavorable HR practices in their organizations. The finding further reveals that CSR’s impact during COVID-19 on employees’ attitudes is moderated by the different levels of CSR importance in employees’ minds. This evidence is significant since HR practices implemented during this crisis need to be identified and framed to understand the effects of CSR on employee commitment and identification. CSR involvement in the pandemic can help managers keep their employees committed to organizations; only if this charity begins from their internal stakeholders first.

## Introduction

The COVID-19 demonstrated a devastating effect on countries worldwide. This condition has sparked a global crisis that has affected every aspect of our lives (Hinojosa et al., 2020). This pandemic outbreak also had a significant adverse effect on the organization’s day-to-day processes. Organizations have implemented various tactics to survive this pandemic (Khan et al., 2021a). Many organizations are entirely out of the game, while some work online, providing vital services to the people impacted by the pandemic (Asghar et al., 2021). The companies adopt various human resource (HR) practices to ensure continuity and financial balance, e.g., cutting employees’ salaries, terminations, and leaving with or without pay (Akingbola, 2020). The hospitality industry is not an exception and is severely affected (Gursoy and Chi, 2020; Khan et al., 2021b). Most restaurants are forced to reduce indoor dining. The travel restrictions dramatically decreased tourist activities and hotel occupancy rates (Baum and Hai, 2020; Giousmpasoglou et al., 2021). Overall, a significant impact on the hospitality industry’s operational viability and growth capability is observed (Fong et al., 2020; Zhang et al., 2020). Nearly two-thirds of restaurant employees have lost their employment, either furloughed or laid off (National Restaurant Association [NRA], 2020).

Due to this outbreak, the employees face a drastic decrease in their income, triggering dramatic changes in their lives. Besides, it adversely impacts their psychological well-being. There is general frustration and anxiety due to an epidemic crisis and income/job uncertainty (Choi et al., 2020). According to a report, layoffs, non-employability, and the adverse impacts of COVID-19 often lead the employees to psychological issues, including stress, anxiety, depression, frustration, and loneliness (World Health Organization [WHO], 2020). Nonetheless, with the increased need for the organization to operate online smoothly, the organizations face resistance and paucity of commitment as they oppose the organizations’ HR policies (Weise and Conger, 2020).

In the alternate world, several organizations agree that promoting corporate social responsibility (CSR) is the best thing to help humanity overcome this pandemic. For example, they develop disinfection and sanitation systems and provide free personal protective equipment (PPEs) devices, food, and ration kits. CSR refers to the organizational policies and initiatives adopted to realize the sustainable development of finance, society, and the environment (Su and Swanson, 2019). For example, social media reported several hotels offering their space to health officials or medical observation following the epidemic. Some of them were turned into rest and rehabilitation centers. In addition, companies took the initiative to safeguard their personnel’s interests, help their suppliers and clients, and contribute through money and goods.

It is widely accepted that CSR positively affects employee reactions in normal circumstances. However, in the current COVID situation, when organizations are using less promising HR practices, this well-designed, socially responsible program may not be considered well-intentioned from the employees’ point of view. Therefore, this study aims to examine whether well-intentioned CSR may lead to unforeseen adverse effects on the overall relationship between CSR and employees’ attitudes.

Prior research highlighted the positive impact of CSR on employees’ commitment in normal circumstances (Shen and Zhu, 2011; Chun et al., 2013). The COVID 19 increased unemployment rates, and we argue that it has impacted the employees’ mental health, leading to poor employee commitment. To achieve the organizational objectives, profitability, and efficiency, committed employees are necessary. Employee commitment may be established if organizations respect their employees’ expectations and needs, use a fair and ethical code of professional conduct, and provide an inclusive work environment, fair salary, friendly policies, and vice versa. However, due to unfavorable HR practices, keeping employees committed will remain one of the top challenges organizations face during the COVID-19. This study proposes that CSR significantly impacts employees’ commitment, but unfavorable HR practices negatively moderate this relationship.

This study uses sensemaking theory to investigate the impact of CSR on employees’ identification and commitment (normative, continuance, and affective) in the presence of unfavorable HR practices (job layoff, cut in salaries) and the perceived importance of CSR. Sensemaking refers to the process where individuals or groups give structure to the unknown (Weick et al., 2005). There are three significant elements of the sensemaking process, i.e., selection, retention, and enactment. As per this approach, employees will pick stimuli from their surroundings, attach meaning to them *via* interpretation, and utilize those meanings to construct a framework of knowing that directs the future course of action (Weick et al., 2005). Thus, sensemaking fosters identity construction, and in return, identity influences the sensemaking process (Weick et al., 2005). In this case, CSR activities will act as positive stimuli for employees when perceived positively, which will foster their identification which further leads to high commitment.

Furthermore, according to Weick (1995), individuals strive to preserve a logical and coherent framework by which they perceive their subjective reality. This structure includes future cues if it remains coherent. Therefore, when employees simultaneously notice the CSR for societal welfare and unfavorable HR practices, this inconsistency will make them believe that the organization is behaving hypocritically. As a result, the positive impact of CSR on employees’ commitment can be lowered or diminished. Therefore, the study also proposes that the importance of CSR will also moderate the impact of CSR on employees’ commitment. Investigating HR practices and CSR importance as moderators is significant because CSR’s positive effects might depend on the cultural context (Pakistan), the belt in these societal practices.

The hospitality business is susceptible to health crises, catastrophes, and other hazards; therefore, this study intends to investigate the CSR phenomenon in the hospitality industry (Jung et al., 2021). Furthermore, the structural aspects of this hospitality industry result in high job insecurity for workers (Robinson et al., 2019; Baum et al., 2020). Several studies investigate the impact of COVID-19 (Qiu et al., 2021) on operational activities (Hao et al., 2020; Jiang and Wen, 2020; Kaushal and Srivastava, 2021) and the integration of technological advancements to deal with CSR crises (Shin and Kang, 2020; Zeng et al., 2020; Kim et al., 2021) in the hospitality industry. However, despite the COVID-19 epidemic, comparatively little research has been conducted on CSR efforts in the hospitality industry and their impact on employees’ attitudes (Aguinis et al., 2020; Gürlek and Kılıç, 2021; Qiu et al., 2021). The focus of prior studies on unexpected occurrences in the hospitality industry mainly studies the consumers and management perspective (Ritchie and Jiang, 2019).

**FIGURE 1:**
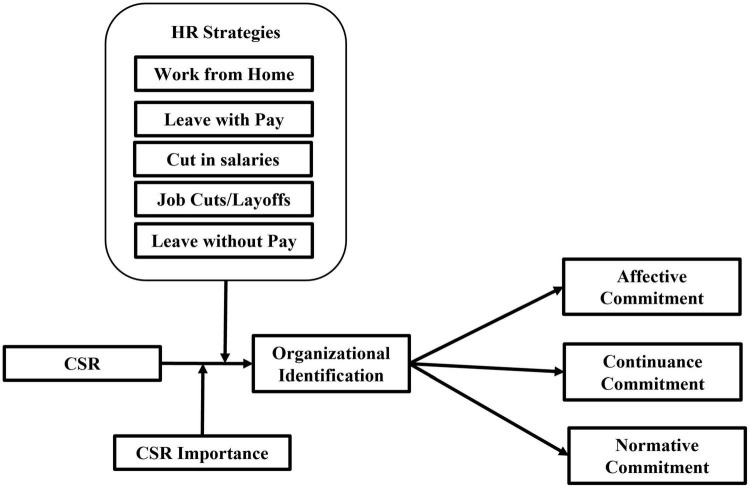
The study model.

A little is known about how such events impact hospitality employees and how the organizational responses to these events might mitigate or temper their impact on hotel employees. Indeed, human resource is crucial to competitiveness in tourism (Croes et al., 2020), so businesses must prioritize employee well-being (Mao et al., 2020). However, it is noticed that hotel industry employees face a drastic decrease in their salaries, layoffs, and triggering dramatic changes in their jobs and their lives. If this question remains unanswered, we will be unable to fully appreciate the role of crisis management in restoring employees’ commitment to the organization. Further, there are inconclusive results about the moderation of CSR importance on employees’ reactions to CSR. Therefore, the study aims to examine the moderating role of CSR importance in CSR and employees’ reactions. The study model is given in Figure 1.

This study responds to the call of research by Aguinis et al. (2020) to examine the effects of HR activities on the COVID-19 CSR response. They argued that despite the explosive studies of recent years connecting CSR with micro research, much of the research focused on theory and areas in OB compared to HRM (Gond et al., 2017, 2020; Gond and Moser, 2021). Thus, we need to know how HR practices are impacted by and, in turn, impact CSR responses to COVID-19. When almost every organization changes its HR policies, the current pandemic gave scholars a chance to integrate theoretical streams and extend investigations into HRM and CSR (Stahl et al., 2020). This research is also relevant since HR procedures implemented during a crisis need to be identified and framed to understand the effects of CSR on employee commitment and identity. It is time for organizations to identify practices not suited to uncertain, volatile, ambiguous, and complex environments.

## Theory and Hypotheses Development

### Sensemaking

Sensemaking in organizations is an ongoing activity through which employees interpret and explain workplace situations. Basu and Palazzo (2008) mention that sensemaking is a trio of cognitive (cogent for engaging in specific actions), linguistic (ways of explaining the rationale to engage in these actions), and conative (behavioral posture to conduct activities with consistency and commitment to sustain the relationships) processes. Sensemaking starts when organizational members face surprising or confusing events, activities, and issues (Weick, 1995). Sensemaking permits individuals to deal with those things by generating rational accounts of the environment that enable their behaviors (Maitlis, 2005). Thomas et al. (1993) said that sensemaking includes environmental scanning, interpretation, and associated reactions of individuals. Most of the research on sensemaking focuses on one actor ignoring the interaction of different actors. Therefore, how the multiple actors in organizations interact to make sense of different activities needs attention (Maitlis, 2005). Weick (1995) argues that without relationships and social roles, the process of sensemaking is impossible to take place. Therefore, we also use a sense-giving perspective in our study besides sensemaking. Sense-giving is “the process of attempting to influence the sensemaking and meaning construction of others toward a preferred redefinition of organizational reality” (Gioia and Chittipeddi, 1991).

### Corporate Social Responsibility and Employees’ Commitment: Mediating Role of Organizational Identification

Commitment is binding of the employees’ behavior and makes it less changeable and predictable (Kiesler and Sakumura, 1966). Organizational commitment is the psychological state of employees about their attachment to the goals and values of the organization due to specific reasons. Most of the literature provide consensus on three dimensions of commitment, namely affective commitment (due to emotional attachment, identification, and involvement), normative commitment (due to socially accepted values and norms), and continuance commitment (due to perceived cost of company change and fewer job opportunities) provided by Meyer and Allen (1991). The affective commitment makes the employees comfortable and satisfied. Continuance commitment depends on the degree of available alternatives and their perceived costs. Finally, normative commitment develops when the organizations do well or pay the reward in advance.

Keeping in view the three processes of sensemaking mentioned above, CSR is the organizational activity that managers do to manage their relationship with stakeholders to achieve the goals of the common good (Basu and Palazzo, 2008). We argue that CSR is a possible antecede to three components of commitment. It keeps the employees satisfied, provides them with a better workplace, and gives them a sense of society’s custodian. Empirical evidence supports this notion. The studies found a positive direct effect of CSR on South Korean casino employees’ organizational commitment and affective organizational commitment (Kim et al., 2016) and South Korean hotel employees (Kim et al., 2017), respectively.

Anyhow, we posit that the above-said relationship is not linear, and some underlying mechanism is needed to explain that relationship. We argue that identification serves as an underlying mechanism as identity construction is one of the basic foundations of sensemaking. Organizational identification (OID) is the initial expression of emotional attachment (Freud, 1960). Identification is shared as the interests of both parties are shared (Podsakoff and MacKenzie, 1989). The identified employees categorize themselves in the definition of their reference organization. It is a powerful emotional characteristic that converts “I” into “we.” The members consider themselves one with the organization, and their decisions are subjective to the mutual and best interest of the organization. They evaluate alternatives differently to satisfy the interest of the organization.

Corporate social responsibility provides the logic to make sense of organizational actions positively. Specifically, CSR, in the context of COVID-19, gives the sense that organizations care for society. During these difficult times, the organization’s social responsibility becomes more critical. When organizations understand their social responsibility and take the initiative, their worth increases for the organizational members. These members make the sense that these activities are valuable for them and society. In addition, the organization’s care of society allows the employees to generate positive psychological states.

Sensemaking starts with a self-conscious sense maker (Weick, 1995). Pratt (2000) states that sensemaking powerfully affects the stakeholders and “construct “their identities; therefore, the sense maker is always in a loop to define the self. Erez and Earley (1993) suggested that the need for self-enhancement affects the sensemaking of the individuals to maintain and develop their identity. The research suggests that both the positive or negative image of the organization affects the individuals’ perceptions, and their identities and self-concepts are partially modified by the image of their organizations (Dutton and Dukerich, 1991). Thus, the sense maker clarifies the organization’s identity and adjusts accordingly. When the sense maker’s values and beliefs are aligned with the organization’s, they want to identify with that organization. Therefore, we argue that CSR positively effects the OID of organizational members.

The organizational members tend to incline to be one with the organization. It makes sense for them to enjoy the basking glory of the organization. In other words, in response to sense-giving, the employees want to identify with that organization to enhance their self-esteem. They categorize themselves in the definition of the organization. The third dimension of the sensemaking perspective suggests that identified employees become emotionally attached to that organization. For some of the members not emotionally attached to the organization, it makes sense to stay with the organization that takes care of the stakeholders in the bad times when people are losing their jobs. It also makes sense that some members attach to the organizations for normative reasons. Identified employees provide support to the organization through their decisions. They try to decide on the interest of the organization. Thus, we propose our first hypothesis.

*H1a: CSR positively impacts employees’ affective commitment through the mediation of Organizational identification.*
*H1b: CSR positively impacts employees’ continuance commitment through the mediation of Organizational identification.*
*H1c: CSR positively impacts employees’ normative commitment through the mediation of Organizational identification.*

### Corporate Social Responsibility and Employees’ Commitment: Moderation of Corporate Social Responsibility Importance

According to Stawiski et al. (2010), a difference in the extent to which CSR is important to employees has a considerable impact on the assessment of significance people assign to advantages generated from CSR. Extending on the same line of study, we claim that employees who firmly believe in the value of corporate social responsibility are more likely to identify with corporations. Employees who value CSR would gain psychologically from realizing that their company is meeting its social and environmental duties. As a result, employees develop a strong identification with the organization; consequently, we propose that CSR is significant to employees helps explain how employees’ views of a firm’s CSR commitment will affect their job-related attitudes. Given this perspective, one may expect CSR relevance to an employee to have a moderating role in explaining why workers’ views of a business’s CSR activities alter employee sentiments toward the organization. As a result, we expect that the effect of employees’ perceived CSR on their identification intensifies when CSR is important to them.

The sensemaking perspective is individual and social (Weick, 1995). Based on that assumption, we argue that different employees make sense of CSR differently, keeping the CSR importance as a contingency on their interpretation and explanation. CSR importance is more like a spectrum of high and low, where employees could be higher, middle, and lower. At the higher end, some employees consider CSR a survival strategy and a tool to gain a competitive advantage. The sensemaking perspective suggests that when employees’ values are highly aligned with the organizational values, their actions will be more positive (Weick, 1995). Therefore, we posit that their commitment to the organization becomes more substantial for those with high CSR importance than those with low CSR importance.

*H2: CSR importance moderates the relationship between CSR and organizational commitment through organizational identification.*

### Moderating Role of HR Practices on Corporate Social Responsibility and Commitment Path

Strategic HR practices play an essential role in developing and maintaining sustainable business operations. However, different organizations adopt different strategies during difficult times. For example, COVID-19 proves a hard time for HR managers to develop and implement contemporary practices in the hospitality industry and align with the company’s overall vision. The most frequent practices that have been widely used are “work from home,” “leave with pay,” “cut in salaries,” “layoff,” “leave without pay,” and unfortunately, “suspension of business.” The sensemaking perspective suggests that unfavorable HR practices can impact the employees’ perception of CSR activities. When employees perceive that their organization is involved in CSR while adopting unfavorable HR practices for internal stakeholders, they might become cynical toward CSR and moderate their attitudes. Weick (1995) suggests that sensemaking is particularly critical within active and unstable contexts like COVID-19 because there is a strong need to develop and sustain consistent understandings that maintain relationships.

Furthermore, the sensemaking perspective suggests that individuals justify reducing the cognitive dissonance when the discrepancy occurs (Weick, 1995). Based on that postulate, we argue that employees try to understand CSR in the context of unfavorable HR practices. As a result, their perception of CSR becomes negative; therefore, they show less commitment toward CSR. The employees cognitively reduce the dissonance by showing less commitment toward the organization and justifying their behavior; therefore, negative HR strategies may not harm their identity and commitment raised due to CSR. Thus, based on the above arguments, we propose that:

*H3: HR practices moderates the relationship between moderated mediated relationship between CSR and organizational commitment through organizational identification.*

## Research Design

Given the complexity of the research question, the primary goal is to reveal the way employees of the hospitality sector experience and react to CSR in the presence of HR practices adopted by these organizations during the COVID-19 crisis. The investigation is thus designed as a causal exploratory study using quantitative research that would enable the researcher to establish causality (Bryman, 2012). There are two significant reasons for selecting the hospitality sector. Although the COVID-19 impacts almost every industry, it directly and massively detrimental influences the hospitality business, damaging the sector throughout Pakistan. In essence, this resulted in the near-immediate closure of this economic sector. Employees are primarily affected by future risks and uncertainties; thus, they were selected as survey participants. Second, hospitality is a labor-intensive industry whereby employees serve as service providers.

**FIGURE 2:**
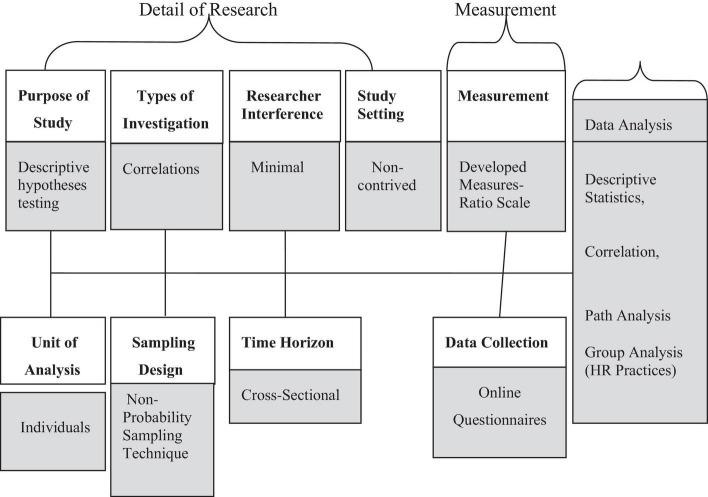
Research design flow chart.

The online survey approach is the most suitable for two reasons among different quantitative research techniques available for exploring such a problem for data collection purposes. First, it is a popular and common strategy in business and management research for exploratory research. It enables the scholar to collect quantifiable data simultaneously with two or more variables. Secondly, in the current pandemic and mandatory lockdown situation, it was not possible to visit the employees at their workplace; an online survey makes it easy to approach many respondents at one time. Figure 2 represents the flow chart for research design of this study.

### Sample and Data Collection

Participants in the study were full-time employees working for at least a year in luxury hotels in Lahore, Pakistan, and non-probability sampling was used to select the study participants. The participants are from Lahore, the second-largest Pakistan city and a tourist destination. Due to the lockdown in Pakistan, it was impossible to visit hotels physically; therefore, HR offices of luxury hotels are contacted online using contacts generated through personal references. After receiving the proper approval, a link to a google form containing survey items is delivered to the HR officers who accepted the request to dispense the questionnaires to different employees. We conducted the survey using Google Forms. Anyone with access to the survey google form link could participate in the study.

The questionnaire consists of three major sections. The first section pertains to a small introductory paragraph and emphasizes the survey’s aim and the respondents’ anonymity and confidentiality. A quick explanation of how to answer the question is also included in the last part of the paragraph. The second section contains participants’ demographic characteristics, such as gender, age, education, and marital status. The final section of the questionnaire addressed all items of the study variables. The hotel HR officer circulates the survey link to their employees to collect data between July and August 2020, when COVID-19 peaked in Pakistan. Over almost a month, 420 answers were recorded online. A total of 28 questionnaires were deemed invalid due to a pattern of choice as respondents selected the same option for more than eight questions in these questionnaires. Finally, 392 surveys were found to be ready for the next phase.

### Measures

Perceived CSR measure is adapted from Ong et al. (2018), consisting of eight items. Due to the novelty of the pandemic, no validated scales to measure CSR activities of hotels in response to COVID-19 are available; therefore, six items are developed by conducting a small study. These six items are our hotel: (1) make an investment in disinfecting and sanitation programs in the COVID affected areas, (2) provide free personal protective equipment (PPE) kits to the frontline healthcare workers during the COVID situation, (3) invest in supporting the district administration in extending healthcare facilities and contributing toward procuring ventilators and other medical equipment during COVID situation, (4) providing immediate relief such as food and ration kits to the workers and affected community during COVID situation, (5) involve in livelihood promotion programs by involving Self Help Groups during COVID situation, (6) involve in health awareness programs during COVID situation. The sample item for Ong et al.’s (2018) scale is “Extent to which your hotel acts responsibly toward people in general.” So, the final scale of perceived CSR consists of 14 items, and a Likert scale of 1–5 is used where one stands for strongly disagree and 5 for strongly agree.

The measure by Meyer and Allen (1991) consists of 24 items to assess continuance, affective, and normative commitment (eight items for each dimension) is used. Mael and Ashforth (1992) devised a six-item scale to assess organizational identification, used in this study. Etheredge (1999) devised a five-item scale to assess the importance of CSR. These three scales are measured using a five-point Likert scale. A small exploratory study is conducted to identify the HR practices to deal with COVID-19. Total six different strategies are identified: work from home, leave with pay, cut in salaries, job cuts/layoffs, leave without pay, and suspension of business. Respondents are asked to select all those practices from these six HR practices adopted by their hotel.

## Data Analysis

The data were screened for missing values and unengaged responses. The data were further analyzed to test the assumptions of normality, linearity, and multicollinearity. The values of Skewness are below one and Kurtosis are below 1.5; thus, we move toward further analysis. In AMOS, we conduct the CFA using latent variables. The values of model fitness fulfill the acceptable range of threshold provided by Hu and Bentler (1999). We also compare our baseline model of six factors against the other alternative models. The values of model fitness measures in Table 1 clearly show that the baseline model performs better than the alternative models.

**TABLE 1:** Confirmatory factor analysis (CFA).

Models	CMIN	DF	CMIN/DF	CFI	SRMR	RMSEA	PClose
6-Factor	2,936.21	1102	2.664	0.935	0.034	0.065	0
5-Factor	4,842.20	1107	4.374	0.867	0.072	0.093	0
4-Factor	7,423.66	1111	6.682	0.775	0.121	0.121	0
3-Factor	9,056.73	1114	8.13	0.717	0.093	0.135	0
2-Factor	10,327.99	1116	9.254	0.672	0.119	0.145	0
1-Factor	11,828.96	1117	10.59	0.618	0.129	0.157	0
							

We calculated the convergent and discriminant validity scores. The results in Table 2 highlight no primary concern of convergent validity because AVE values are above 0.5 and larger than MSV values. Further, discriminant validity is not concerned as the square root of the variance of variables is greater than the correlation among the other variables. After the model validity, we conducted the Pearson bivariate correlation analysis and calculated the Cronbach Alpha to test the reliability of the scales. The values in Table 3 show that variables are correlated in the desired direction. Furthermore, the alpha values in diagonal show that scales are reliable as all the values are above the threshold value of 0.70.

**TABLE 2:** The results of convergent and discriminant validity analysis.

	CR	AVE	MSV	CSR	AC	CC	NC	IMP	OID
CSR	0.96	0.73	0.54	**0.86**					
AC	0.98	0.88	0.68	0.66***	**0.94**				
CC	0.98	0.85	0.61	0.74***	0.64***	**0.92**			
NC	0.98	0.84	0.61	0.71***	0.75***	0.78***	**0.92**		
IMP	0.92	0.71	0.30	−0.55***	−0.15**	−0.21***	–0.06	**0.84**	
OID	0.97	0.83	0.68	0.71***	0.83***	0.61***	0.73***	−0.15**	**0**.**913**

n = 392, ***p < 0.001, and **p < 0.01

**TABLE 3:** Mean, standard deviation, correlation, and reliability.

	Mean	SD	CSR	OID	AC	CC	NC	IMP
CSR	2.54	0.99	**0.97**					
OID	3.61	1.36	0.73**	**0.97**				
AC	3.40	1.41	0.67**	0.84**	**0.98**			
CC	3.36	1.32	0.75**	0.62**	0.65**	**0.98**		
NC	3.52	1.08	0.72**	0.74**	0.77**	0.79**	**0.98**	
CSR IMP	3.25	0.82	−0.57**	−0.16**	−0.16**	−0.22**	–0.07	**0**.**92**
HRPr	2.63	1.52	0.04	0.00	–0.03	0.07	0.01	0.00

n = 392, **p < 0.01.

*OID, organizational identification; CC, continuance commitment; CSR IMP, CSR importance; AC, affective commitment; HRPr, human resource practices; NC, normative commitment.*

**FIGURE 3:**
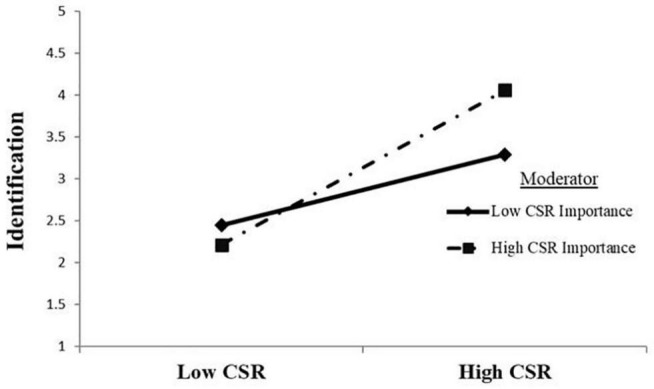
The moderation of corporate social responsibility (CSR) importance on CSR and Identification.

To test the study hypotheses, we conducted path analysis using AMOS and result are provided in Table 4. The first hypothesis about the mediation of identification between CSR and commitment is supported. There is significant positive relationship between CSR and (a) affective commitment (β = 0.406, *p* < 0.001), (b) continuance commitment (β = 0.077, *p* < 0.05) and normative commitment (β = 0.244, *p* < 0.001). We developed an interaction term of CSR and CSR importance to test our second hypothesis. The result shows a significant effect of the interaction term (β = 0.45, *p* < 0.001) on identification. The result supports the second hypothesis. Figure 3 presents that CSR importance strengthens the positive relationship between CSR and identification.

**TABLE 4:** The results of path analysis of the combined model.

Direct effect on dependent variables		*B*	*P*-Value
CSR	Identification	0.408	0.031
Identification	Affective commitment	0.722	0.001
Identification	Continuance commitment	0.137	0.015
Identification	Normative commitment	0.435	0.001
**Indirect effect on dependent variables**			
CSR	Affective commitment	0.406	0
CSR	Continuance commitment	0.077	0.008
CSR	Normative commitment	0.244	0
**Moderation of CSR importance**			
CSR × CSR importance	Identification	0.45	0.001

The third hypothesis of the study is about the moderation of HR practices on the CSR and commitment mechanism. The moderation results are provided in Table 5. Findings show that work from home decrease the strength of the indirect positive effect of CSR on affective commitment (from 0.406^**^ to 0.287*) and normative commitment (0.244^***^ to 0.188*) while the positive relationship with continuance commitment (0.077^**^ to 0.058) becomes insignificant. On the other hand, the leave with pay increases the strength of the indirect positive effect of CSR on affective commitment (from 0.406 to 0.551), continuance commitment (0.077^**^ to 0.144), and normative commitment (0.244^***^ to 0.577*). Cut in salaries increases the strength of the indirect positive effect of CSR on affective commitment (from 0.406^**^ to 0.443*) and normative commitment (0.244^***^ to 0.255*), while the positive relationship with continuance commitment (0.077^**^ to 0.045) become insignificant.

**TABLE 5:** Moderation of HR practices.

	The indirect effect of CSR			The direct effect of CSR			
Moderator	*AC*	*CC*	*NC*	*OID*	*AC*	*CC*	*NC*
WFH	0.287*	0.058	0.188*	0.408*	0.187**	0.647***	0.387***
LWP	0.551***	0.144**	0.577***	1.207**	0.246	0.664*	0.322**
CIS	0.443**	0.045	0.255**	0.624**	0.155**	0.706***	0.432***
LO	0.253	0.08	0.161	0.283	−0.014	0.504**	0.26*
LWOP	0.357	0.075	0.193	0.412	0.053	0.641***	0.438***

n = 392, *p < 0.05, **p < 0.01, and ***p < 0.001.

*WFH, work from home; LWP, leave with pay; CIS, cut in salaries; LO, layoffs; LWOP, leave without pay.*

On the other hand, is the case of leave without pay (from 0.406 to 0.357), the indirect positive effect of CSR on affective commitment (from 0.406^**^ to 0.287*), normative commitment (0.244^***^ to 0.193), and continuance commitment (0.077^**^ to 0.05) remains positive but become insignificant. Similarly, in the case of employees’ layoff, the indirect positive effect of CSR on affective commitment (from 0.406^**^ to 0.253*), normative commitment (0.244^***^ to 0.161), and continuance commitment (0.077^**^ to 0.08) also remains positive but become insignificant. Therefore, it means organizational CSR does not have any impact on commitment for those employees who observed unfavorable HR practices of their organization.

## Discussion

This study investigated the impact of CSR on hotel employees’ identification and commitment during the COVID-19 pandemic by using the sensemaking perspective. In addition, the moderation of CSR importance and HR practices is also explored. Following are the main findings of the study. First, CSR by hotels has a positive impact on their employees’ commitment (affective, continuance, and normative) through the mediation of organizational identification. In this pandemic, when organizations fight for survival, their involvement in CSR will let employees believe that organizations are socially responsible and have genuine care toward society. This positive evaluation enhances the organizational worth in the eyes of its members. It creates a sense of oneness in employees’ minds for their organizations, therefore becoming more identified. Finally, the identified employees become committed to their organization. It makes sense for them to stay with the organization that takes care of the stakeholders in the bad times when people are losing their jobs. This conclusion is coherent with prior study findings that suggested that CSR positively influences employees’ job engagement and organizational citizenship behavior (Kim et al., 2017; Wang et al., 2020).

The second main finding of this study is about which HR practices during pandemic moderates the relationship between CSR and organizational commitment through organizational identification. We find that work from home, leave with pay, and cut in salaries positively impact the relationship between CSR and employees’ identification and commitment. These HR strategies increase CSR’s positive impact on employees’ commitment because employees might feel grateful for being a part of the organization even in difficult situations. Whereas employees’ layoff and leave without pay are insignificant on CSR and commitment relationship. We argue that when employees are laid off and receive leave without pay, it triggers dramatic changes in their lives. They get stressed, which ultimately leads to a change in their commitment level with the organization. The employees may judge organizational CSR actions as symbolic and immoral. Therefore, organizations could face resistance and paucity of commitment on the part of employees as they oppose the organizations’ HR practices.

Further, the study finding reveals that the positive impact of CSR on employees’ commitment is moderated by CSR importance. This relationship strength is based on the extent to which CSR is important for an individual employee. This finding suggests that organizational CSR efforts do not equally impact all individuals; instead, their personal preferences for CSR impact their attitudinal response toward CSR. This conclusion contradicts the findings of Chaudhary (2020), who found that the importance of CSR did not affect how CSR influences employee behavior. However, this conclusion is consistent with Turker’s (2009) and Peterson’s (2004) research, where a higher effect of CSR on organizational commitment is reported in those employees who strongly believe in the importance of CSR.

### Study Implications

The new organizational and social environment created by COVID-19 opens a new study path for expanding our knowledge of behavioral CSR. One of the critical gaps in the current scenario is understanding how employees feel about CSR when their incomes are reduced, they are laid off, or their occupations are drastically altered. This research has several significant consequences for academics and practitioners. First, this study leads to a better understanding of behavioral CSR by looking at the impact of CSR in response to COVID-19 on employees’ identification and commitment. In contrast, its in-house employees are subjected to harsh HR policies. We extended the prior CSR research that validates that CSR impacts employees’ identification and commitment by providing evidence that this relationship will become insignificant if the organization fails to devise fair strategies for its employees. Second, this research suggests that such well-intentioned CSR in a pandemic crisis leads to decreased employee identification and commitment. Thus, this research contributes to the employees’ resistance to CSR literature. Third, the study provides empirical evidence on how employees interpret, give meaning, and react to CSR differently depending on organizational HR policies and individual characteristics.

Fourth, the study contributes to the research about contingency variables on CSR-employee outcome research by validating that unfavorable HR practices and CSR importance are significant contingency variables on this link. Finally, researchers take a unique position to evaluate the impact of the crisis recession on employees who work in the hospitality sector in developing economies overly dependent on tourism revenue (Novelli et al., 2018).

For practitioners, first, the study suggests that CSR involvement in the pandemic can help managers keep their employees committed to organizations. The study reveals that CSR initiatives in pandemic situations will improve employees’ attitudes toward the organization. A recent study reveals that employees are willing and excited to work for an organization involved in meaningful work (Goh and Baum, 2021). However, poor HR procedures in stressful situations might negatively impact workers’ sentiments toward their employers and decrease the positive impact of CSR on commitment. Because CSR is connected to sensemaking, informing employees about CSR participation for external society and poor HR practices for internal stakeholders leads to reduced employee identification and commitment. Therefore, the hospitality industry needs a balance through adequate measures to be socially responsible toward society and their employees to keep their employees identified and committed.

Second, organizations should emotionally comfort their employees, as personal qualities might change depending on the circumstances. For example, suppose strategies such as staff downsizing, salary cuts, or unpaid leave are used as a reactive management tool. In that case, each decision must be convincingly communicated to employees. Furthermore, any preparations for securing jobs from governmental authorities must be communicated to employees to offer them the best option during the epidemic. Indeed, previous research (Bui et al., 2019) has shown that open and frequent communication can improve employee performance.

Third, the business must devise a contingency plan with mutual involvement of employees. Earlier studies (Vinodkumar and Bhasi, 2010; Pham et al., 2020) demonstrate that involving employees in strategic insights and choices make them feel at ease, secured, engaged and increase corporate positive attitudes and behavior. Organizations should support and respect those who have brought valuable ideas. Sharing initiative moments should be joyful and engrained in the culture to prevent potential emotional stress.

### Limitations and Future Direction

Despite its significant insights, the current study has some constraints that should be noted. First, the study’s cross-sectional nature, causal relationships between CSR, organization identity, and commitment could not be inferred. As a result, a follow-up longitudinal study in the post-COVID-19 period would give additional confirmation of such associations. Second, this research focused solely on self-reported data and employee perspectives. Finally, this study was conducted with Pakistani personnel, and it may have some cross-cultural limitations. Thus, from the perspective of institutional theory, more research should be performed in other cultures from both developing and developed countries with diverse social security systems, cultural values, labor regulations, and other contrasting factors.

## Data Availability Statement

The raw data supporting the conclusions of this article will be made available by the authors, without undue reservation.

## Ethics Statement

The studies involving human participants were reviewed and approved by the Ethical Committee of Lahore Business School, The University of Lahore. The patients/participants provided their written informed consent to participate in this study.

## Author Contributions

All authors listed have made a substantial, direct, and intellectual contribution to the work, and approved it for publication.

## Conflict of Interest

The authors declare that the research was conducted in the absence of any commercial or financial relationships that could be construed as a potential conflict of interest.

## Publisher’s Note

All claims expressed in this article are solely those of the authors and do not necessarily represent those of their affiliated organizations, or those of the publisher, the editors and the reviewers. Any product that may be evaluated in this article, or claim that may be made by its manufacturer, is not guaranteed or endorsed by the publisher.
